# Preparation of carrier-free astaxanthin nanoparticles with improved antioxidant capacity

**DOI:** 10.3389/fnut.2022.1022323

**Published:** 2022-09-28

**Authors:** Fei Yu, Jiaxin Chen, Zizhan Wei, Pingchuan Zhu, Qing Qing, Bangda Li, Huimin Chen, Weiying Lin, Hua Yang, Zhongquan Qi, Xuehui Hong, Xiao Dong Chen

**Affiliations:** ^1^Medical College, Guangxi University, Nanning, China; ^2^State Key Laboratory for Conservation and Utilization of Subtropical Agro-Bioresources, College of Life Science and Technology, Guangxi University, Nanning, China; ^3^Guangxi Key Laboratory of Electrochemical Energy Materials, School of Chemistry and Chemical Engineering, Institute of Optical Materials and Chemical Biology, Guangxi University, Nanning, China; ^4^The Fourth People's Hospital of Nanning, Nanning, China; ^5^Department of Gastrointestinal Surgery, Zhongshan Hospital of Xiamen University, Xiamen, China; ^6^Suzhou Key Lab of Green Chemical Engineering, School of Chemical and Environmental Engineering, College of Chemistry, Chemical Engineering and Materials Science, Soochow University, Suzhou, China

**Keywords:** astaxanthin, carrier-free, antioxidant, food industry, nanoparticles

## Abstract

Astaxanthin (AST), a red pigment of the carotenoids, has various advantageous biological activities. Nevertheless, the wide application of AST is restricted due to its poor water solubility and highly unsaturated structure. To overcome these limitations, carrier-free astaxanthin nanoparticles (AST-NPs) were fabricated through the anti-solvent precipitation method. The AST-NPs had a small particle size, negative zeta potential and high loading capacity. Analysis of DSC and XRD demonstrated that amorphous AST existed in AST-NPs. In comparison with free AST, AST-NPs displayed enhanced stability during storage. Besides, it also showed outstanding stability when exposed to UV light. Furthermore, the antioxidant capacity of AST-NPs was significantly increased. *In vitro* release study showed that AST-NPs significantly delayed the release of AST in the releasing medium. These findings indicated that AST-NPs would be an ideal formulation for AST, which could contribute to the development of novel functional foods.

## Introduction

Astaxanthin (AST) is a lipid-soluble and red keto-carotenoid that has been separated from sundry microorganisms, phytoplankton, marine animals and seafood (1–4). It is commonly applied in the food, beverages, aquaculture, cosmetics and pharmaceutical industries due to its excellent antioxidant activity (5). Massive studies have demonstrated that the antioxidant activity of AST is 10 times higher than β-carotene and 100 times more powerful than vitamin E (6, 7). However, AST is highly unsaturated and decomposes easily when exposed to light, heat and oxygen during storage. In addition, its poor water solubility, pungent odor and instability have hampered its wide application in human nutrition (8).

Different methods have been explored to solve these issues, such as β-cyclodextrin complexes, liposomes and polymeric nanospheres (9). For example, the stability of AST was significantly improved when using MCC and CMC-Na as wall materials (10). AST nano-dispersion was prepared using WPI and PWP through an emulsification-evaporation technique and showed increased bioavailability (11). Besides, it has been reported that the antioxidant capacity of AST-nanodispersions was much greater than that of free AST (12, 13). Nevertheless, these preparation process usually require some chemical cross-linking agent (aldehydes), which may have certain security concerns (14). Furthermore, the design and synthesis of multi-component delivery systems are usually time-consuming and complicated. To avoid possible toxicity from carriers or problems related to biodegradation, carrier-free drug delivery systems (DDS) have been recently developed (15, 16). The nanostructures of carrier-free DDS were aggregated by pure drugs and could maximize the loading capacity of hydrophobic drug molecules. Meanwhile, many carrier-free nanoparticles were successfully prepared by anti-solvent method (17). These carrier-free DDS are speculated to self-assemble *via* hydrophobic interactions (π-π stacking) or hydrogen bonds in the structures of the molecules. On this basis, we wondered whether the carrier-free AST nanoparticles could also be designed.

**Figure 1:**
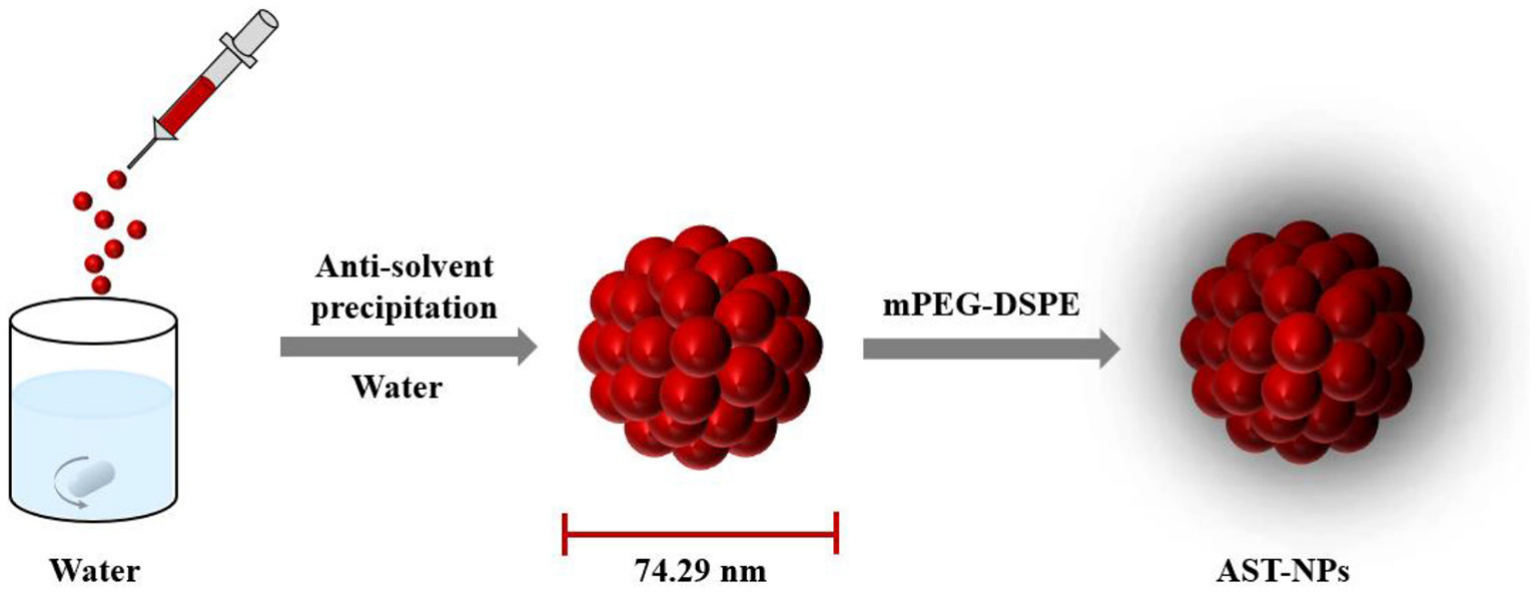
Schematic illustration of the AST-NPs.

The aim of our study was to fabricate carrier-free astaxanthin nanoparticles (AST-NPs) through the anti-solvent precipitation method (Figure 1). The physicochemical properties of AST-NPs were systematically measured. Meanwhile, the photostability and storage stability of AST-NPs were also determined to study the potential of industrial application. Additionally, XRD and DSC were used to assess the crystalline nature and thermal property of AST-NPs, respectively. Finally, the *in vitro* release behavior of AST-NPs as well as its antioxidant activity was also evaluated. These results indicated that AST-NPs could simultaneously improve the stability and antioxidant capacity of AST, which would extend the use of lipophilic nutraceuticals (AST) in foods.

## Materials and methods

### Materials

Astaxanthin (AST) (pure > 97%) was bought from Aladdin Industrial Corporation (Shanghai, China). ABTS and potassium persulfate were purchased from Shanghai Macklin Biochemical Co., Ltd. (Shanghai, China). All other solvents including dimethyl sulfoxide (DMSO) and ethanol were supplied by Sinopharm Chemical Reagent Co., Ltd (Shanghai, China).

### Preparation

AST-NPs were fabricated by the anti-solvent method. AST-DMSO solution was obtained by dissolving AST in DMSO through sonication treatment for 1 min. 250 μL of AST-DMSO solution was dropwise injected into 10 mL of DI water and then mPEG-DSPE was added under stirring condition. Finally, the AST-NPs were fabricated after being stirred for 2 h at 40 °C.

### Characterization of AST-NPs

The mean particle size of AST-NPs was determined by DLS. The morphology of AST-NPs was examined by TEM (HT-7700, Hitachi High-Technologies Corporation, Japan). 200 μL of sample was dropped to a copper gird, which was dried by a filter paper. The samples were subjected to TEM after drying. The surface morphology of AST-NPs was also examined under a SEM (Hitachi, SU8220, Japan). The gold spray treatment was carried out on samples before measurement.

### Loading capacity

The UV-Vis spectroscopy was used to determine LC of AST-NPs. Briefly, the AST-NPs were transferred into a centrifuge tube and then centrifuged. The precipitation was collected and mixed with DMSO-water solution. The absorbance of samples was determined by a UV-Vis spectrophotometer to calculate the concentration of AST. A standard calibration curve of AST in DMSO-water solution was previously obtained: Y = 0.1834X - 0.009 (R^2^ = 0.9996), where Y and X refer to the absorbance and concentration of AST, respectively. Finally, LC was investigated according to the following equation:


(1) $$\begin{array}{l} LC\;\left(\%\right)=\frac{Total\;AST-free\;AST}{Total\;amount\;of\;nanoparticles}\times \;100\% \end{array}$$

### XRD patterns

The XRD patterns of AST, mPEG-DSPE and AST-NPs powders were recorded using an X-ray diffractometer (Rigaku D/MAX 2500V). The XRD patterns were recorded from 5 to 60° at a speed of 10 °/min.

### DSC measurements

The measurement of DSC (STA 449 F3, NETZSCH) was carried out to characterize the thermal property of samples. Briefly, the sample was placed in a sealed aluminum pan. The DSC curves were scanned from 50 to 300 °C.

### Storage stability

The samples of free AST and AST-NPs were transferred to the transparent tube and then stored at room temperature in a light-proof cabinet for 72 h to investigate the stability behavior.

### Photostability study

The photostability of AST-NPs was assessed by the UV-Vis spectroscopy. In brief, the samples were transferred to transparent container and then exposed to UV light for 70 min. The samples were collected at certain times (0, 10, 20, 30, 40, 50, 60 and 70 min). The retention rate of AST was calculated:


(2) $$\begin{array}{l} Retention\;rate\;of\;AST\;\left(\%\right)=\frac{A}{A_{0}}\times \;100\% \end{array}$$

where A and A_0_ represent the absorbance at different time points and the initial absorbance of AST, respectively.

### *In vitro* release study

The release rate of AST-NPs *in vitro* was studied by the dialysis method. The samples of free AST and AST-NPs were poured inside dialysis bags, respectively. Then, the dialysis bags were placed into PBS buffer with Tween-80 (0.1%) and kept under 100 rpm for 240 min in a constant temperature incubator shaker (MQT-60, Shanghai Minquan Instrument Co., LTD). At pre-determined intervals (30, 60, 90, 120, 150, 180, 210 and 240 min), 4 mL of sample inside the dialysis bags was collected. The absorbance of each sample was determined to estimate the percentage of the released drug.

### Antioxidant activity

ABTS stock solution and potassium persulfate solution were mixed in equal volumes. ABTS working solution can be obtained by diluting with ethanol. Then, 3 mL of ABTS working solution was mixed with an equal volume of sample solution for 10 min. All of the samples at 734 nm were determined by a UV-Vis spectrophotometer.

## Results and discussion

### Preparation and characterization of AST-NPs

**Figure 2:**
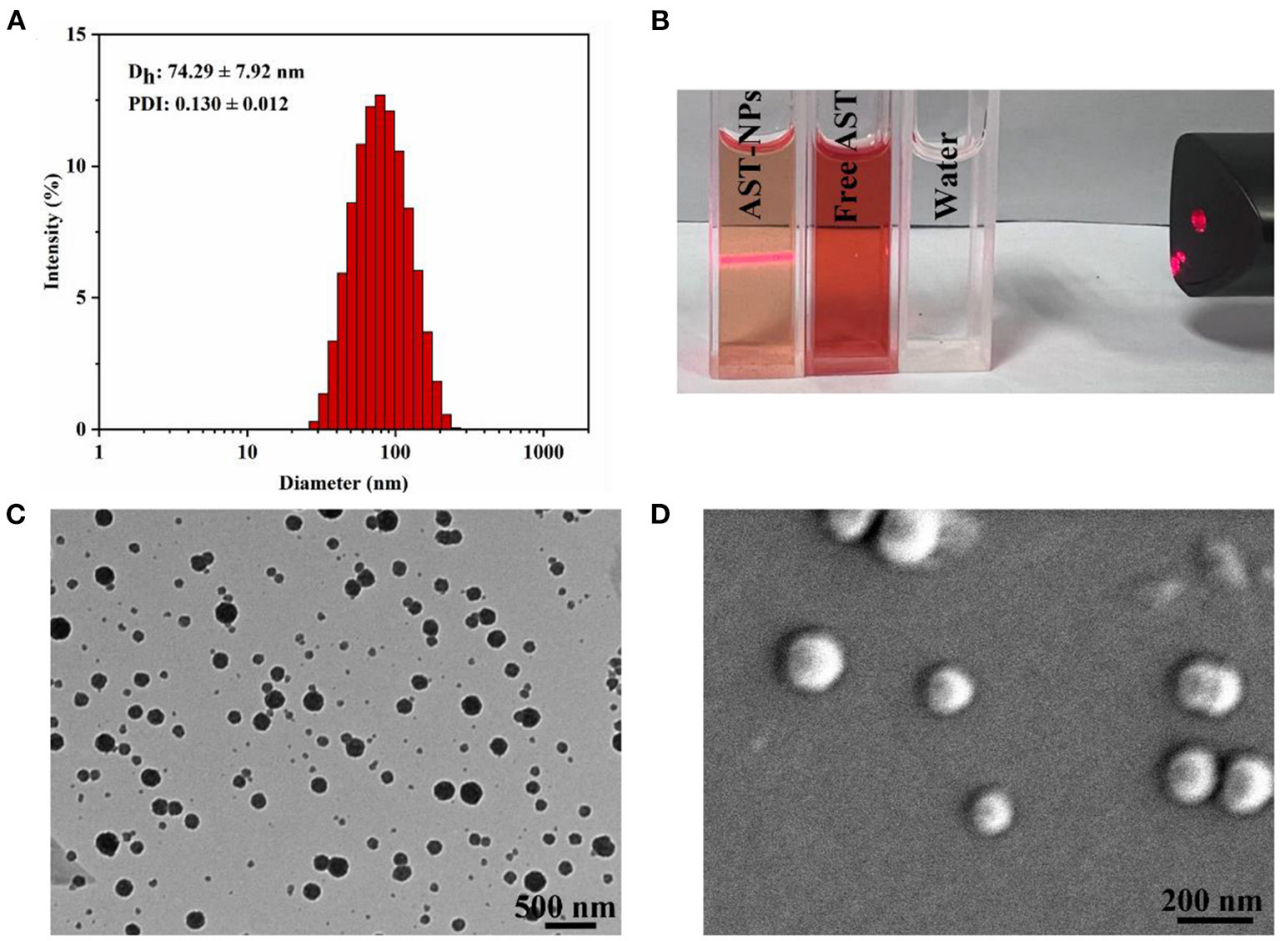
**(A)** Particle size and PDI of AST-NPs. **(B)** Tyndall effect of AST-NPs. **(C)** TEM image and **(D)** SEM image of AST-NPs.

As depicted in Figure 2A, the obtained AST-NPs presented a nearly monodisperse particle size distribution with a mean diameter of 74.29 ± 7.92 nm (PDI = 0.130 ± 0.012). In this study, the zeta potential of AST-NPs was −14.4 ± 2.96 mV, which was responsible for the stability of the nanoparticles. In addition, an obvious Tyndall effect could be observed *via* a laser (Figure 2B), which suggested the formation of nanoparticles (18). The morphological characteristics of the AST-NPs were investigated by TEM and SEM. The TEM image displayed uniformly dispersed AST-NPs with spherical structure (Figure 2C) and the SEM photo also showed that the nanoparticles were well-dispersed without any aggregation (Figure 2D). The LC (94.57 ± 0.70%) of AST-NPs was significantly improved than that of other carrier-based DDS (mostly <10%). This phenomenon might be attributed to that the stable interaction forces could be formed between AST molecules without the help of any carrier.

### XRD analysis

**Figure 3:**
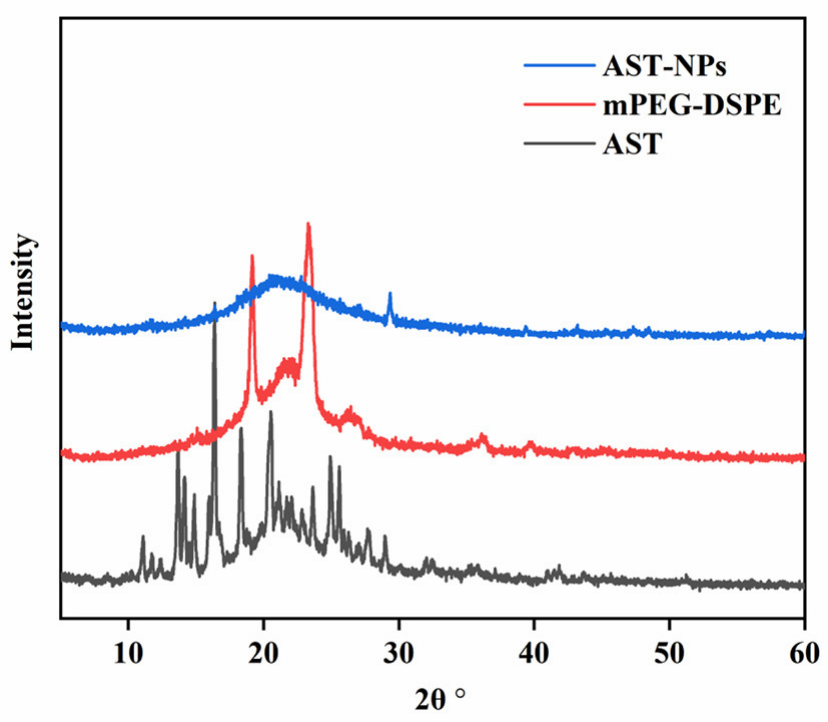
XRD patterns of AST-NPs, mPEG-DSPE and free AST.

The stability and solubility of functional components are related to their crystalline state (19). XRD measurements were performed on free AST, mPEG-DSPE and AST-NPs to obtain information about the crystalline state (Figure 3). Generally, the more peaks in XRD patterns of samples mean the higher degree of structural crystallites (20). The XRD pattern of free AST indicated multiple distinct characteristic peaks at 11.1, 13.7, 16.38, 18.36, 20.52 and 25.6°, which were related to its crystalline nature (21). By contrast, these sharp peaks in the diffractogram of AST-NPs were disappeared. This phenomenon indicated that the AST was embedded in the nanoparticles and transformed the crystal form into an amorphous form. Typically, the modification of a crystalline nature through nano-dimension can be an ideal way for elevating the solvability of drugs, which may be advantageous for the application of AST (19).

### DSC analysis

**Figure 4:**
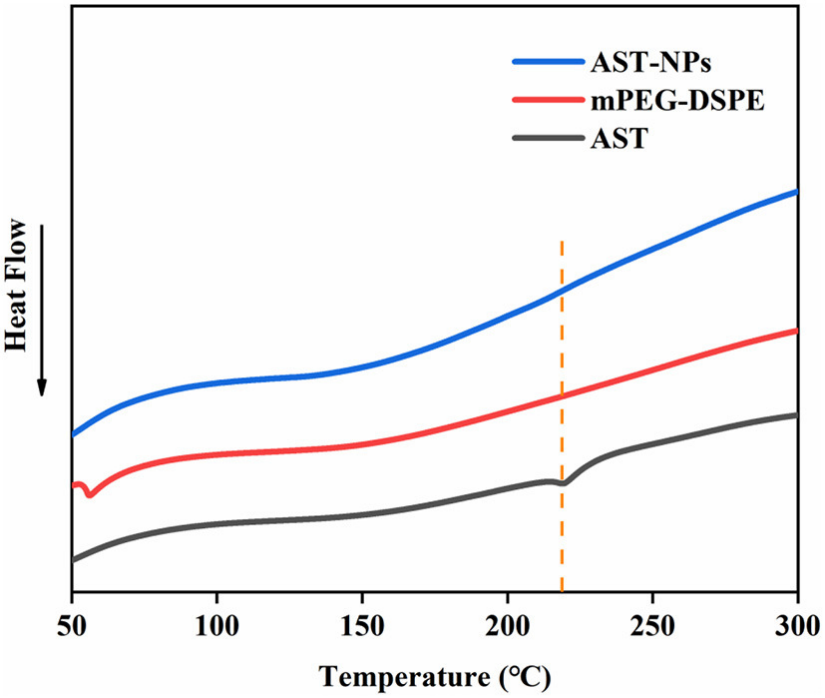
DSC thermograms of AST-NPs, mPEG-DSPE and free AST.

DSC analysis could qualitatively reflect changes in physical state of drugs, relying on the changes of endothermic peaks (22). DSC thermograms of free AST, mPEG-DSPE and AST-NPs were shown in Figure 4. The free AST exhibited an endothermic peak near 218 °C corresponding to its melting point, which showed that AST was present in a crystal form (23). However, the melting endothermic peak of AST was not visible in the DSC spectrum of AST-NPs, which indicated that the AST was in an amorphous form rather than in a crystalline form. In addition, it also indicated that the AST was successfully encapsulated into AST-NPs. Unlike crystalline AST, the dissolution of amorphous AST required less energy to diffuse, making it have better solubility (24). These results were consistent with the findings of XRD analysis.

### Storage stability

**Figure 5:**
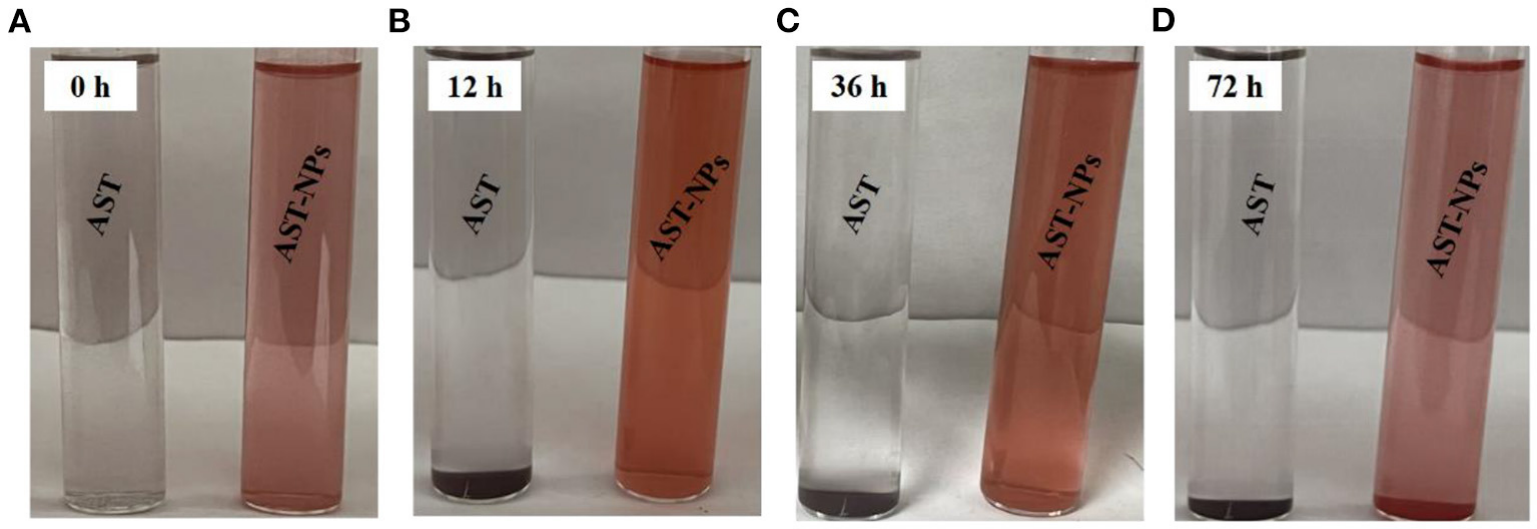
The visual appearances of free AST and AST-NPs at 25°C at different time points.

For AST-NPs, it is important that they have excellent stability during storage time (25). For this reason, we recorded the changes in the physical properties of the AST-NPs dispersion over time and compared them to the aqueous solution of free AST. Obvious differences between free AST and AST-NPs in anti-aggregation ability were observed after the storage for 72 h at room temperature in the dark. Specially, as shown in Figures 5A–D, no obvious visual differences were observed in the nano-dispersion (72 h). In contrast, visual observations of free AST indicated the formation of sediment at the bottom of the test tubes over time, which suggested that free AST molecules aggregated during storage. Meanwhile, the appearance and turbidity of the AST-NPs dispersion remained relatively unchanged after being stored at 25 °C for 36 h. In fact, only slight color fading and a small amount of sediment were observed after 72 h. This could be attributed to the high hydrophilicity and favorable charge characteristics, which could protect AST-NPs against aggregation during storage.

### Photostability assay

**Figure 6:**
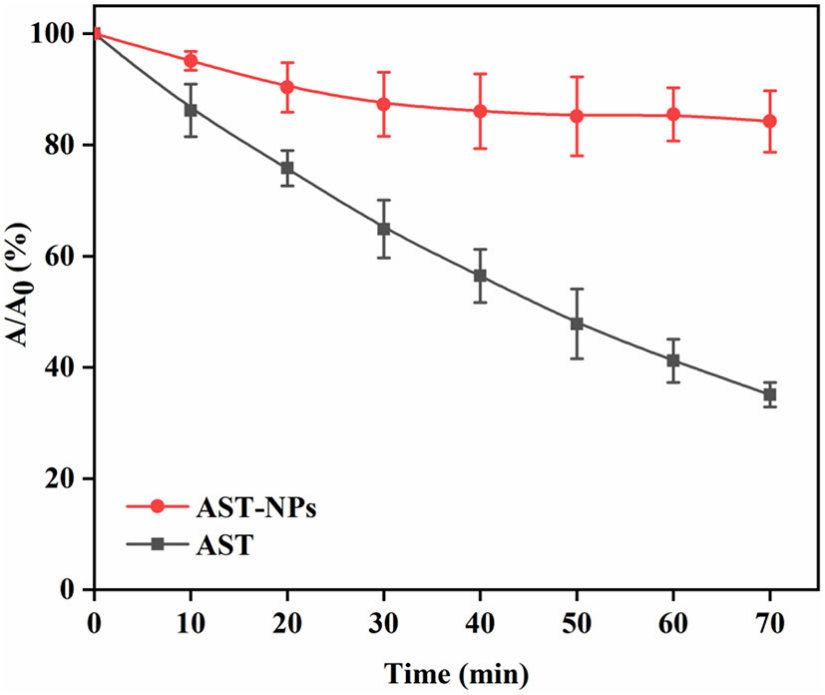
Retention rate of AST-NPs and free AST under UV light.

Photochemical degradation of AST could lead to its loss of biological activity (26). The photostability of free AST or AST-NPs was investigated by exposing them to UV light. As can be seen in Figure 6, comparing to free AST, AST-NPs were more stable against UV light due to the physical barrier of nanoparticles. On the contrary, the degradation of free AST varied linearly when it was continuously exposed to UV light. It should be noted that, following with illumination, the retention rate of AST in all samples was decreased. After the first 30 min of UV treatment, 87.30 ± 5.74% of AST-NPs suspension was unchanged. At the same time, 64.87 ± 5.18% of free AST was retained. After the whole lighting period of 70 min, the retention rate of free AST was 35.07 ± 2.20%. In contrast, 84.23 ± 5.53% of AST-NPs survived due to the protective effect from UV light afforded by encapsulation. The observation suggested that AST-NPs considerably improved the photostability of AST, which would be constructive to the application of AST in the food industry.

### Release property of AST-NPs

**Figure 7:**
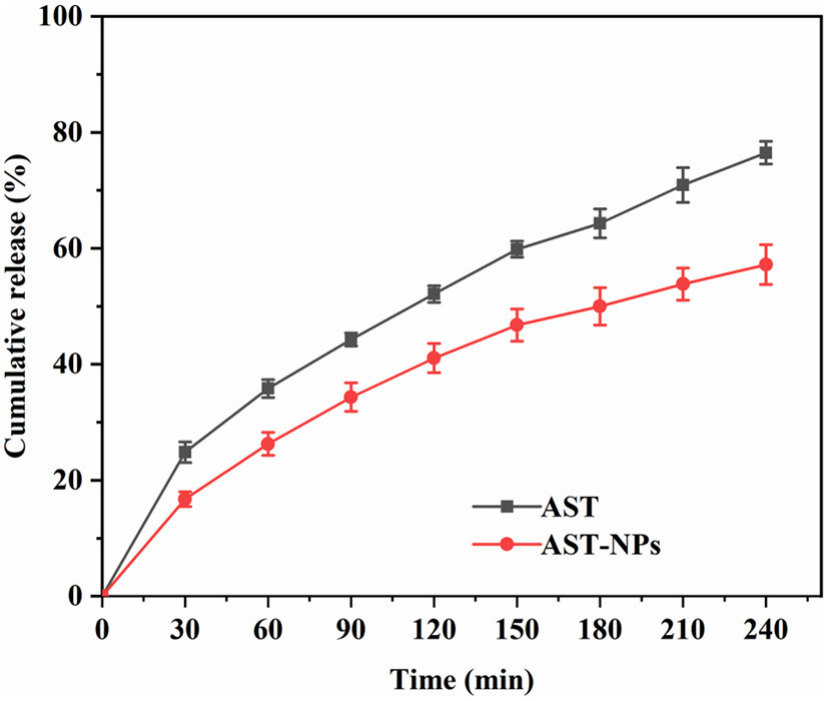
*In vitro* release profiles of free AST and AST-NPs.

An ideal drug delivery system should release the drug in a controlled way as well as possess excellent loading capability (27). Figure 7 depicted *in vitro* release profiles of free AST and AST-NPs. For AST-NPs, a burst release was presented within 30 min in PBS buffer, which might be due to the loss of weakly adsorbed AST molecules on or near the surface of the AST-NPs. After the burst release, the drug release of AST-NPs was slowed down. At 240 min, the release of free AST was 76.48 ± 1.96% whereas the drug release of AST-NPs was 57.20 ± 3.44%. Compared with free AST, the release rate of AST-NPs decreased significantly, which indicated that the AST-NPs achieved controlled release effect. This phenomenon could be attributed to the fact that various non-covalent interactions were existed between AST molecules. The slow and steady release of AST-NPs is essential for providing the body with a continuous supply of AST (28). These results indicated that AST-NPs would be an effective delivery system for AST.

### Antioxidant activity

**Figure 8:**
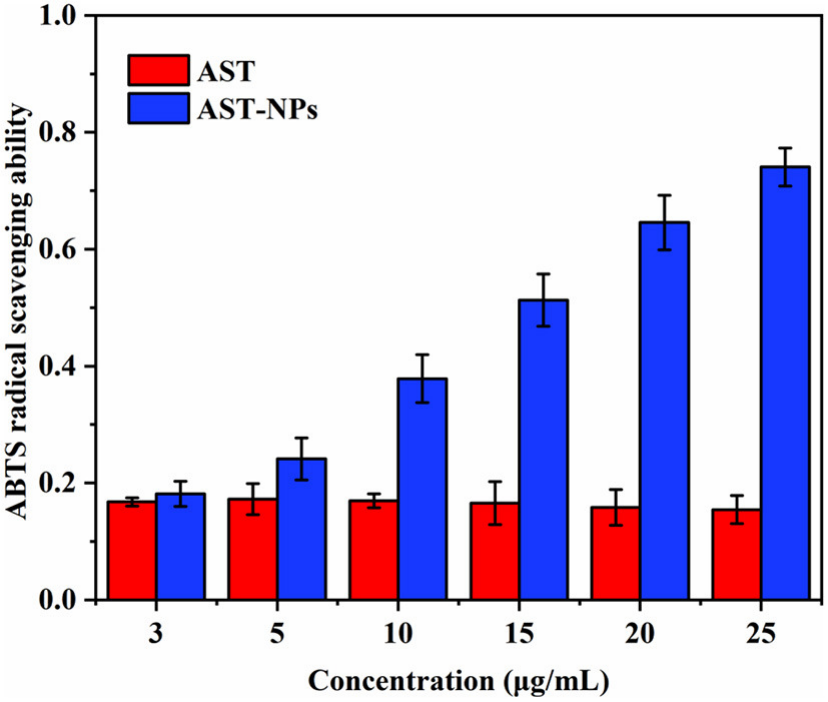
ABTS radical scavenging ability of free AST and AST-NPs.

The high antioxidant activity of AST has been reported owing to the keto groups and hydroxyl groups in the β-ionone ring (29). One of the convenient ways to evaluate the antioxidant activity is to react the AST with ABTS radical (30, 31). The ABTS radical scavenging rate of free AST and AST-NPs was shown in Figure 8. For all samples of AST-NPs, with the increasing of the concentrations, the ABTS scavenging effect increased in a concentration-dependent manner. As a lipophilic compound, the free AST showed relatively low scavenging activity at a wide range of concentrations (3–25 μg/mL) due to its poor solubility (32). At the concentration of 25 μg/mL, the ABTS radical scavenging activity of free AST was 15.45 ± 2.40%. In contrast, the ABTS radical scavenging activity of AST-NPs at equal concentration was 74.07 ± 3.26%, which indicated that the AST-NPs could lead to the increased ABTS radical scavenging activity. The reason for the enhancement of antioxidant activity of AST-NPs may be due to its improved dispersibility, which increased the amount of AST molecules to interact with ABTS radical. Therefore, the AST-NPs would be an efficient way to improve the antioxidant capacity of AST.

## Conclusion

In summary, the AST-NPs were successfully fabricated through the anti-solvent precipitation method. The obtained AST-NPs exhibited extremely high loading capacity (94.57 ± 0.70%), small average size (74.29 ± 7.92 nm) and uniform morphology. The AST-NPs increased the solubility of AST in water and improved its physicochemical properties. The AST-NPs, as a delivery system for AST, not only exhibited excellent ABTS radical scavenging activity but also had a relatively sustained release effect in the releasing medium. This study proved the feasibility of AST-NPs as a delivery system. Therefore, the AST-NPs would provide a new sight for the development of functional foods.

## Data availability statement

The original contributions presented in the study are included in the article/supplementary material, further inquiries can be directed to the corresponding authors.

## Author contributions

FY and JC: methodology, investigation, and data collection. PZ, WL, and HY: provided assistance in the use of the instrument. ZW: data analysis and writing-original draft. QQ, HC, and BL: critical revision and editing of the manuscript. FY, ZQ, XH, and XDC: conceptualization, review, and funding acquisition. All authors contributed to the article and approved the submitted version.

## Funding

This work was supported by the National Natural Science Foundation of China (82003300, 81771721, 81971505, 21877048, 22077048, and 21672083), the National Key R&D Program of China (2018YFA0108304), the Natural Science Foundation of Guangxi Province (2020GXNSFBA297131, AD21220026, 2021GXNSFDA075003, and AD21220061), and the start-up grant from Guangxi University (A3370051003 and A3040051003).

## Conflict of interest

The authors declare that the research was conducted in the absence of any commercial or financial relationships that could be construed as a potential conflict of interest.

## Publisher's note

All claims expressed in this article are solely those of the authors and do not necessarily represent those of their affiliated organizations, or those of the publisher, the editors and the reviewers. Any product that may be evaluated in this article, or claim that may be made by its manufacturer, is not guaranteed or endorsed by the publisher.

## Abbreviations

AST: Astaxanthin
CMC-Na: Carboxymethyl cellulose sodium
MCC: Microcrystalline cellulose
WPI: Whey protein isolation
PWP: Polymerized whey protein
PVP: Poly-vinylpyrrolidone
DLS: Dynamic light scattering
PDI: Polydispersity index
mPEG-DSPE, 1: 2-distearoyl-sn-glycero-3-phosphoethanolamine-N-[methoxy(polyethyleneglycol)]
DSC: Differential scanning calorimetry
XRD: X-ray diffraction
TEM: Transmission electron microscopy
SEM: Scanning electron microscopy
LC: Loading capacity
ABTS, 2: 2'-azino-bis (3-ethylbenzothiazoline-6-sulfonic acid).
